# Extracellular components in enteroaggregative *Escherichia coli* biofilm and impact of treatment with proteinase K, DNase or sodium metaperiodate

**DOI:** 10.3389/fcimb.2024.1379206

**Published:** 2024-05-29

**Authors:** Viktoria Van Nederveen, Angela Melton-Celsa

**Affiliations:** ^1^ Department of Microbiology and Immunology, Uniformed Services University, Bethesda, MD, United States; ^2^ Henry M. Jackson Foundation for the Advancement of Military Medicine, Inc., Bethesda, MD, United States

**Keywords:** biofilm, enteroaggregative *E. coli* (EAEC), extracellular biofilm matrix, enzymatic treatment, aggregative adherence fimbriae (AFF), bacterial adhesion, extracellular DNA (eDNA), confocal microscopy

## Abstract

Enteroaggregative *E. coli* (EAEC) is a major cause of diarrhea worldwide. EAEC are highly adherent to cultured epithelial cells and make biofilms. Both adherence and biofilm formation rely on the presence of aggregative adherence fimbriae (AAF). We compared biofilm formation from two EAEC strains of each of the five AAF types. We found that AAF type did not correlate with the level of biofilm produced. Because the composition of the EAEC biofilm has not been fully described, we stained EAEC biofilms to determine if they contained protein, carbohydrate glycoproteins, and/or eDNA and found that EAEC biofilms contained all three extracellular components. Next, we assessed the changes to the growing or mature EAEC biofilm mediated by treatment with proteinase K, DNase, or a carbohydrate cleavage agent to target the different components of the matrix. Growing biofilms treated with proteinase K had decreased biofilm staining for more than half of the strains tested. In contrast, although sodium metaperiodate only altered the biofilm in a quantitative way for two strains, images of biofilms treated with sodium metaperiodate showed that the EAEC were more spread out. Overall, we found variability in the response of the EAEC strains to the treatments, with no one treatment producing a biofilm change for all strains. Finally, once formed, mature EAEC biofilms were more resistant to treatment than biofilms grown in the presence of those same treatments.

## 1 Introduction

Enteroaggregative *E. coli* (EAEC) is a pathotype of diarrheagenic *E. coli* (DEC) characterized by a stacked-brick adherence (Nataro et al., 1987; Vial et al., 1988). EAEC is a cause of acute and chronic diarrhea worldwide (Flores and Okhuysen, 2009; Hebbelstrup Jensen et al., 2014, 2016), and a common cause of travelers’ diarrhea (Adachi et al., 2001; Hebbelstrup Jensen et al., 2014; Porter et al., 2017; Barrett and Brown, 2018; Guiral et al., 2019; Walters et al., 2020). EAEC is associated with acute diarrhea in children (Nataro et al., 2006; Hebbelstrup Jensen et al., 2014, 2016; Modgil et al., 2021; Kabir et al., 2022). In developing countries EAEC infection is associated with failure-to-thrive (FTT) (Steiner et al., 1998; Hebbelstrup Jensen et al., 2016; Rogawski et al., 2017; Das et al., 2021). FTT is characterized by a rate of weight gain below normal (Jaffe, 2011). Beyond stunting adult height, FTT can lead to learning difficulties and reduced adult earning potential (Jaffe, 2011).

Currently, there is no prevention for EAEC infection (Flores and Okhuysen, 2009), though travelers’ diarrhea is treated with azithromycin or rifaximin (DuPont et al., 2001; Infante et al., 2004; Connor et al., 2012; Riddle et al., 2017; Tribble, 2017). Perhaps due to widespread antibiotic use, EAEC worldwide have high rates of antibiotic resistance (Mendez Arancibia et al., 2009; Aslani et al., 2011; Guiral et al., 2019; Elhadi et al., 2020; Eltai et al., 2020).

EAEC is characterized by its aggregative adherence and ability to create a biofilm (Nataro et al., 1987, 1992; Albert et al., 1993; Czeczulin et al., 1997). The aggregative adherence fimbriae (AAF) are important for both adherence to epithelial cells and other surfaces (Nataro et al., 1987; Nagy et al., 2016; Petro et al., 2020; Schiller et al., 2021), and biofilm formation (Nataro et al., 1992; Czeczulin et al., 1997; Boisen et al., 2012). One of the five genetically different AAF types (AAF1 to AAF5, also called AAF/I to AAF/V) are found in all “typical” EAEC (Savarino et al., 1994; Boisen et al., 2008). AAF1 was shown to be important for colonization and biofilm formation on spinach and abiotic surfaces (Nagy et al., 2016). AAF2 and AAF4 were significantly associated more with diarrhea cases than asymptomatic children in India (Modgil et al., 2021). A study on EAEC-mediated diarrhea in Iranian children found that isolates positive for *agg4A* (AAF4), *pic*, and *sepA* formed a stronger biofilm *in vitro* than strains without the three genes (Nezarieh et al., 2015). Biofilm formation on the intestinal mucosal is thought to be important for EAEC to cause illness (Vial et al., 1988; Tzipori et al., 1992; Nataro et al., 1996; Nataro and Kaper, 1998; Hebbelstrup Jensen et al., 2014).

As part of a biofilm, many pathogens have an extracellular matrix made up of proteins, carbohydrates, and extracellular DNA (eDNA) (Sanchez-Torres et al., 2010; Flemming et al., 2023). These extracellular components have important roles for antibiotic tolerance and transmission of antibiotic resistance, inflammation, and immune evasion (Tetz et al., 2009; Tetz and Tetz, 2010; Zhao et al., 2013; Gunn et al., 2016; Sharma et al., 2016). Targeting the extracellular components of a biofilm can improve treatment effectiveness (Jiang et al., 2020). The prototype EAEC strain 042 (AAF2) is known to make a polysaccharide-rich coat (Borgersen et al., 2018), but the overall composition of the EAEC biofilm is unknown.

In this work, we characterized the extracellular matrix of biofilms formed by recently isolated EAEC strains. We selected two strains for each of the five genetically distinct AAF types to provide a cross section of different EAEC strains. We defined EAEC for this work as having both *aggR* [EAEC virulence gene regulator (Nataro et al., 1994; Morin et al., 2010, 2013; Boisen et al., 2019)] and the genes for production of an AAF. We hypothesized that the selected EAEC would have an extracellular matrix with different combinations of protein, eDNA, and carbohydrate that would correlate to AAF type and/or to level of biofilm formation.

## 2 Results

### 2.1 Biofilm formation by EAEC of each AAF type

**Figure 1:**
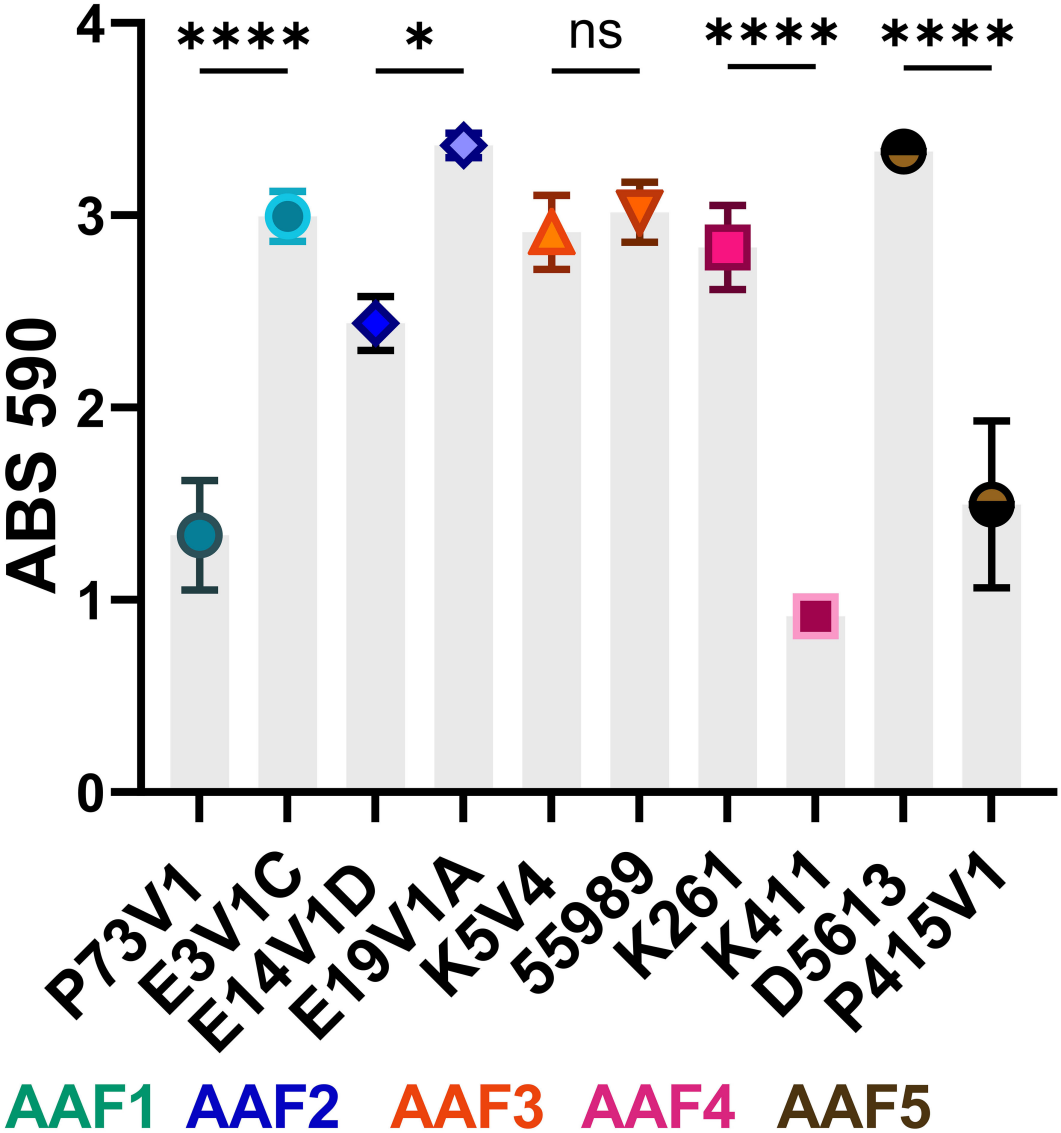
Biofilm staining of EAEC from all five AAF types. The symbols indicate the mean staining from at least three biological replicates. Error bars indicate standard error of the mean (SEM). Differences in biofilm staining were tested for each AAF type by one-way analysis of variance (ANOVA) with Šídák correction. *P ≤ 0.05; ****P ≤ 0.0001; ns — not significant, *P* > 0.05.

The utilized EAEC strains have different sets of virulence factors (**Table 1**), and a wide range of biofilm staining as measured with crystal violet (**Figure 1**). When we compared EAEC with the same AAF type, we found that AAF type did not dictate the level of biofilm staining. For example, while P73V1 (AAF1) had the lowest biofilm staining, E3V1C (AAF1) was among the highest for biofilm staining. Additionally, K261 and K411, both AAF4, had a large difference in mean biofilm staining. Only the AAF3 strains had no statistical difference in biofilm staining. Taken together, these results show that AAF type alone is not predictive of the level of biofilm staining that will be measured for an EAEC strain.

**Table 1:** Virulence factor genes^#^ of all wild-type strains used in this study.

Strain	Aggregative Adherence Fimbriae					Adhesins						Dispersin		Regulators			*aaiC* (T6SS effector)	SPATES/Toxins					
	aggA AAF1	aafA AAF2	agg3A AAF3	agg4A AAF4	agg5A AAF5	hra1	Curli	AIDA-I	E. coli common pilus	type I fimbriae	long polar fimbriae	aap	aatA	aar	aggR	eilA		astA	pic	pet	sat	sepA	sigA
**P73V1**	**+**						**+**	**+**	**+**	**+**		**+**	**+**	**+**	**+**		**+**	**+**	**+**	**+**	**+**		
**E3V1C**	**+**					**+**	**+**	**+**	**+**	**+**	**+**	**+**	**+**	**+**	**+**		**+**		**+**		**+**		
**E14V1D**		**+**				**+**	**+**	**+**	**+**		**+**	**+**	**+**	**+**	**+**	**+**	**+**	**+**	**+**	**+**			
**E19V1A**		**+**				**+**	**+**	**+**	**+**		**+**	**+**	**+**	**+**	**+**	**+**	**+**	**+**	**+**	**+**			
**K5V4**			**+**				**+**	**+**	**+**	**+**		**+**	**+**	**+**	**+**		**+**		**+**				
**55989**			**+**			**+**	**+**		**+**	**+**	**+**	**+**	**+**	**+**	**+**		**+**	**+**	**+**				**+**
**K261**				**+**			**+**	**+**	**+**	**+**		**+**	**+**		**+**		**+**		**+**		**+**	**+**	
**K411**				**+**			**+**	**+**	**+**	**+**	**+**	**+**	**+**		**+**		**+**					**+**	
**D5613**					**+**		**+**		**+**		**+**	**+**	**+**	**+**	**+**		**+**	**+**	**+**		**+**	**+**	
**P415V1**					**+**		**+**	**+**	**+**			**+**	**+**	**+**	**+**		**+**	**+**	**+**		**+**		

^#^Whole genome sequence contigs published in (Boisen et al., 2020; Petro et al., 2020) and for strain 55989 NCBI Reference Sequence NC_011752.1 and NZ_CP028304.1 were queried with the Center for Genomic Epidemiology’s online Virulence Finder, Serotype Finder, and Plasmid Finder platforms, or with Clone Manager version 9 (RRID: SCR_014521). In this table, only virulence genes that one or more strains were positive for are shown.

### 2.2 Staining of EAEC biofilms to assess extracellular matrix composition

**Figure 2:**
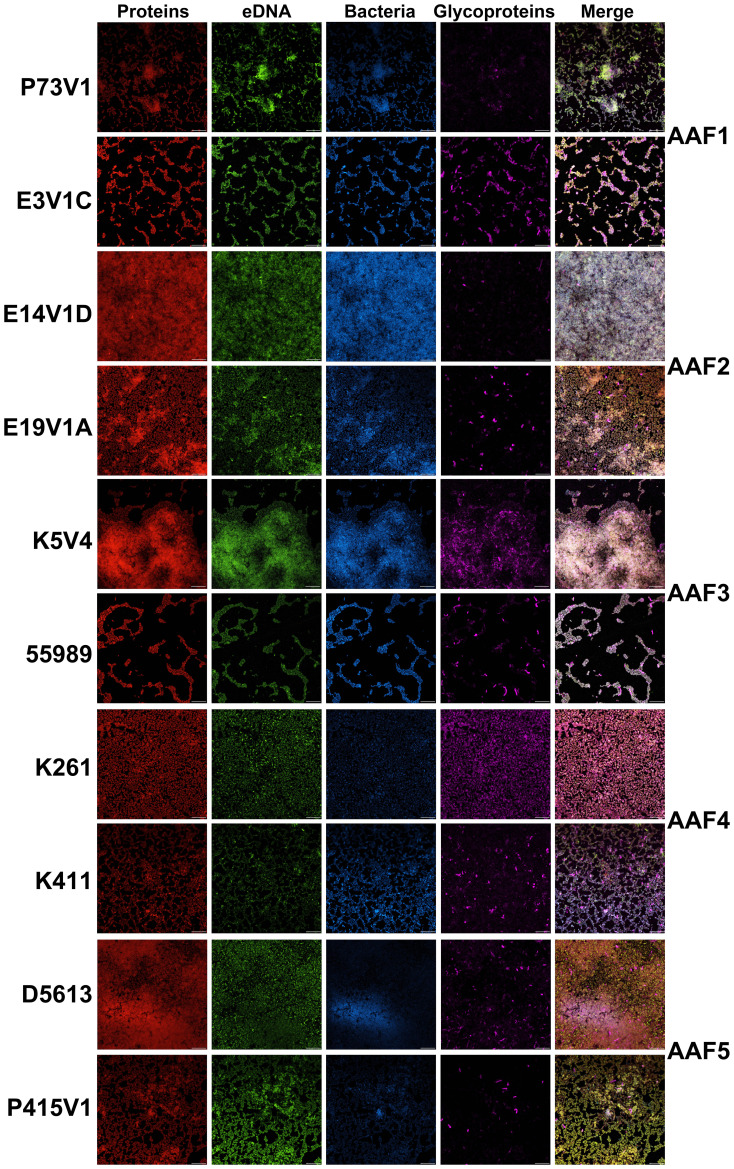
EAEC biofilms visualized with fluorescent stains. Biofilms grown on glass disks for 25 hours were fixed and incubated with Filmtracer Sypro Ruby Biofilm Matrix (red) for most classes of proteins, TOTO-1 iodide (green), an eDNA stain, wheat germ agglutinin (WGA) conjugate (violet) for glycoproteins, and Hoechst (blue) as a counterstain. Representative images from two experiments are shown with the same magnification. Scale bar, 10µm. Full size images for this figure are available through FigShare doi:10.6084/m9.figshare.25761510.

To examine the extracellular components of the EAEC biofilm, we tested fluorescent dyes that target the specific components that typically make up a bacterial biofilm: protein, eDNA, and carbohydrate, **Figure 2**. Our first observation after the staining was that all of the biofilms looked similar, featuring strong staining for protein (red), eDNA (green) and minimal staining with the wheat germ agglutinin (WGA) for glycoprotein (violet). We did not see a large difference in the appearance of biofilm with the different strains we tested. We noted that WGA only stained a proportion of the cells in biofilms from each of the EAEC, a result that suggests that only some cells in the biofilms have a coating that binds the lectin. Bacteria within the biofilm that did not stain with the WGA may have a coating that would react with a different lectin. One EAEC strain, K411 (AAF4), stained brightly with the WGA (violet), but was not as well stained by the TOTO-1 (green) or Sypro Ruby (red) stains. Finally, for the most part, the EAEC in the biofilms adhered in one to two layers and the extracellular matrix appeared to be a coating around each cell, as seen in the pseudo three-dimensional (3D) composite images (**Supplementary Figure 1**). Longer incubation times did not result in biofilms with more layers.

### 2.3 Treatment of biofilm extracellular matrix and fluorescent stains

**Figure 3:**
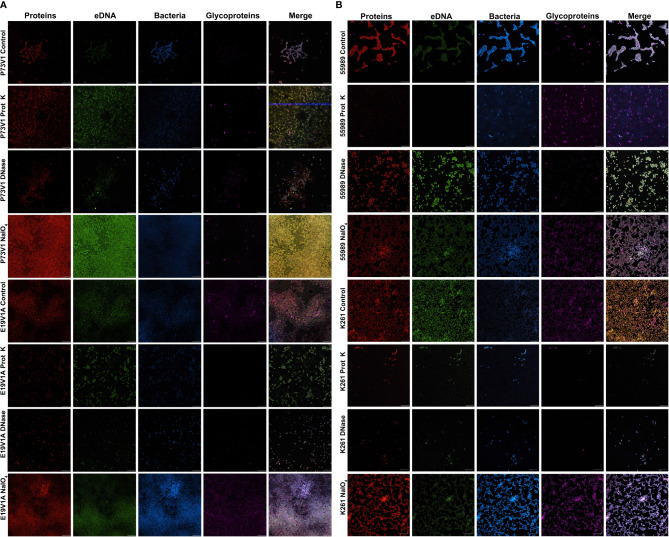
Images of biofilms grown with proteinase K, DNase or sodium metaperiodate followed by staining with fluorescent dyes. **(A)** P73V1 (AAF1) and E19V1A (AAF2) biofilms. **(B)** 55989 (AAF3) and K261 (AAF4) biofilms. Biofilms were grown for 25 hours with media that contained 1 mg/ml proteinase K (prot K) 1 mg/ml DNase I (DNase), or 7.5 mM sodium metaperiodate (NaIO_4_) or a vehicle control (PBS). After growth fixed biofilms were stained identically to **Figure 2**. Representative images from two experiments. Scale bar, 10µm. Full size images for this figure are available through FigShare doi:10.6084/m9.figshare.25761576 and doi:10.6084/m9.figshare.25761612.

Since the biofilms were highly homogeneous when visualized with fluorescent staining, we decided to treat the biofilms from four of the EAEC strains with agents to disrupt the biofilm, and then stain as before. We chose proteinase K, which is a broad-spectrum, nonspecific protease; DNase, which nonspecifically digests single- and double-stranded DNA; and sodium metaperiodate, which is a chemical that cleaves sugars of carbohydrates, including glycoprotein polysaccharides, into reactive aldehyde groups. All of these treatments have been used previously to target other bacterial biofilms (Hall-Stoodley et al., 2008; Tetz et al., 2009; Tetz and Tetz, 2010; Beltrame et al., 2015; Shukla and Rao, 2017; Lim et al., 2019; Deng et al., 2022; Schaffer et al., 2023). Our results were less dramatic than we had expected (**Figure 3**); we generally did not see reduced staining for the targeted components, therefore, we did not do these experiments in the other strains in the study. Yet for some treatments, such as E19V1A (AAF2) with DNase (**Figure 3A**), and E19V1A (AAF2), 55989 (AAF3), and K261 (AAF4) with proteinase K, there appeared to be fewer EAEC in the image from the treated disk than in the control (**Figure 3**). In addition, for P73V1 (AAF1), sodium metaperiodate treatment seemed to increase the overall staining. Finally, 55989 (AAF3) appeared to be more dispersed after DNase treatment. Taken together, these results suggested that the treatments did have an impact on some biofilms, perhaps by altering the matrix in ways that are not easily detected.

### 2.4 Growth of the biofilm with DNase, sodium metaperiodate, or proteinase K in media

**Figure 4:**
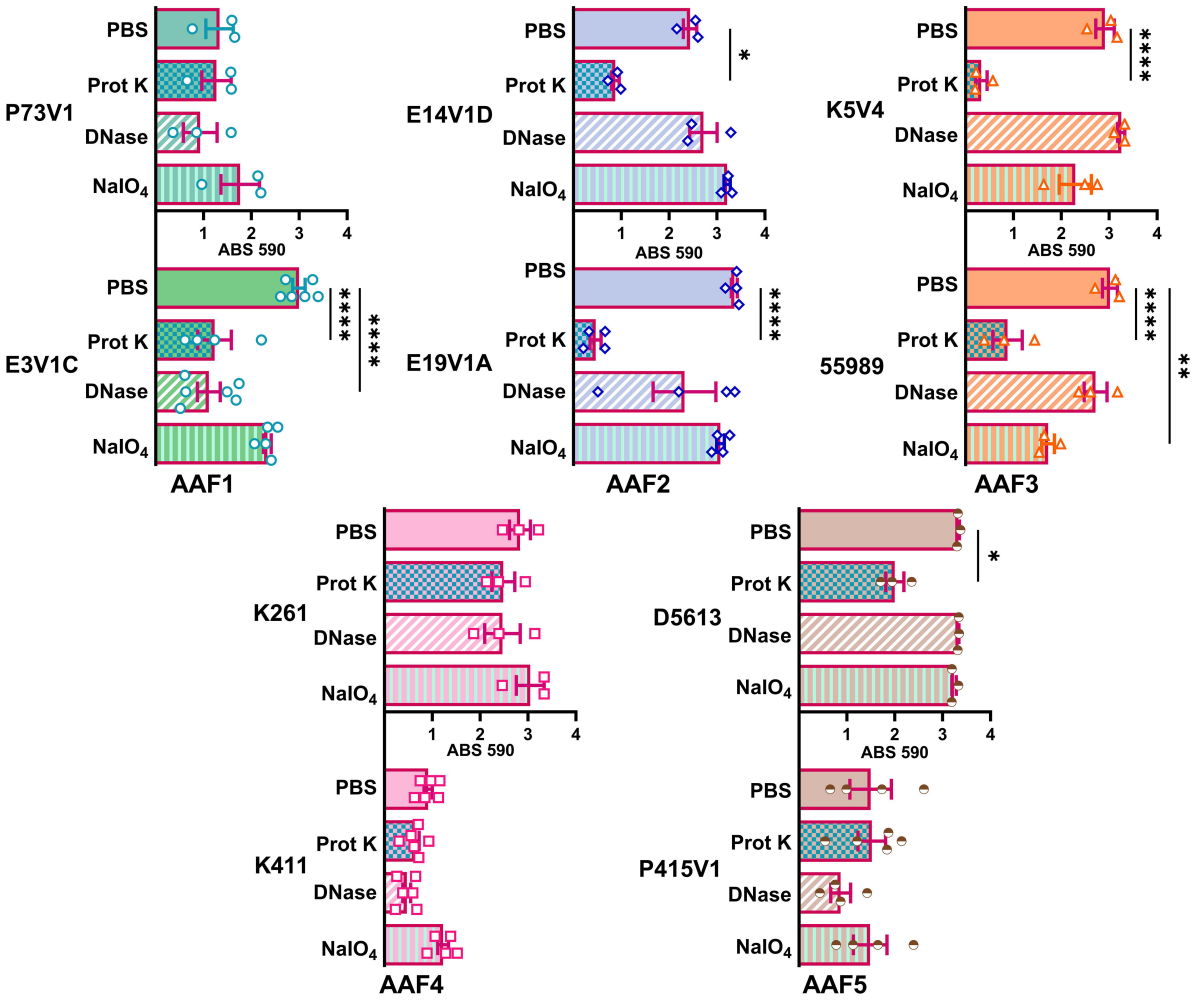
Quantitative staining from biofilms grown in the presence of the indicated treatment. Biofilms were grown for 18 hours with 1 mg/ml proteinase K (prot K) 1 mg/ml DNase I (DNase), or 7.5 mM sodium metaperiodate (NaIO_4_) or a vehicle control (PBS) in the media. Each dot represents the mean of four technical replicates. The overall mean is shown by the bars. Error bars indicate SEM. Significance was tested by one-way ANOVA with Šídák correction. *P ≤ 0.05; **P ≤ 0.01; ****P ≤ 0.0001.

**Figure 5:**
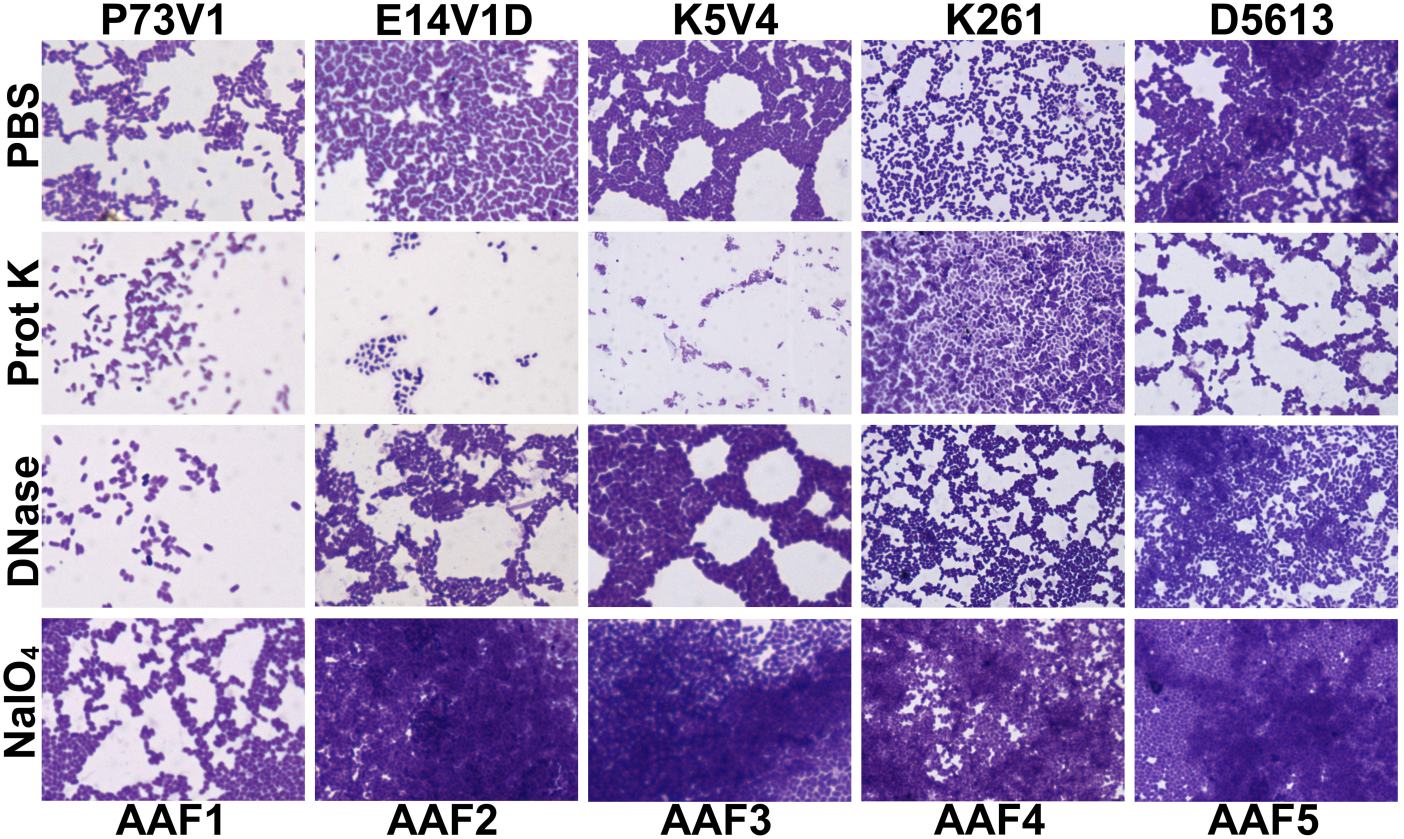
Crystal violet-stained biofilms after concurrent in-media treatment. Biofilms were grown for 18 hours with media supplemented with 1 mg/ml proteinase K (prot K) 1 mg/ml DNase I (DNase), or 7.5 mM sodium metaperiodate (NaIO_4_) or a vehicle control (PBS). The biofilm disks were stained with crystal violet and imaged at 100x. Representative images were selected from two independent experiments.

We next quantified the effect of treating the developing biofilms on biofilm staining with crystal violet (**Figure 4**). Overall, proteinase K treatment reduced biofilm staining in six of the ten strains. DNase treatment at the start of biofilm growth reduced staining for only two of ten strains in a statistically significant way, but we observed a non-significant reduction in biofilm staining for several other strains. Many strains, P73V1 (AAF1), E14V1D (AAF2), and K261 & K411 (AAF4) had a non-significant increase in biofilm staining with the addition of sodium metaperiodate (**Figure 4**). Therefore, we assessed treated biofilms by microscopy in a subset of strains. The microscopy results indicated that the apparent increase in staining after sodium metaperiodate treatment may be driven by the cells of the biofilm being more spread out, and the biofilm may be thicker (**Figure 5**). Finally, for strains E14V1D (AAF2), K5V4 (AAF3), and D5613 (AAF5), we saw a reduction in quantitative biofilm staining with proteinase K treatment and an apparent break-up of the biofilm in the disk images (**Figures 4** and **5**).

### 2.5 Treatment of mature biofilms with DNase, proteinase K, or sodium metaperiodate

**Figure 6:**
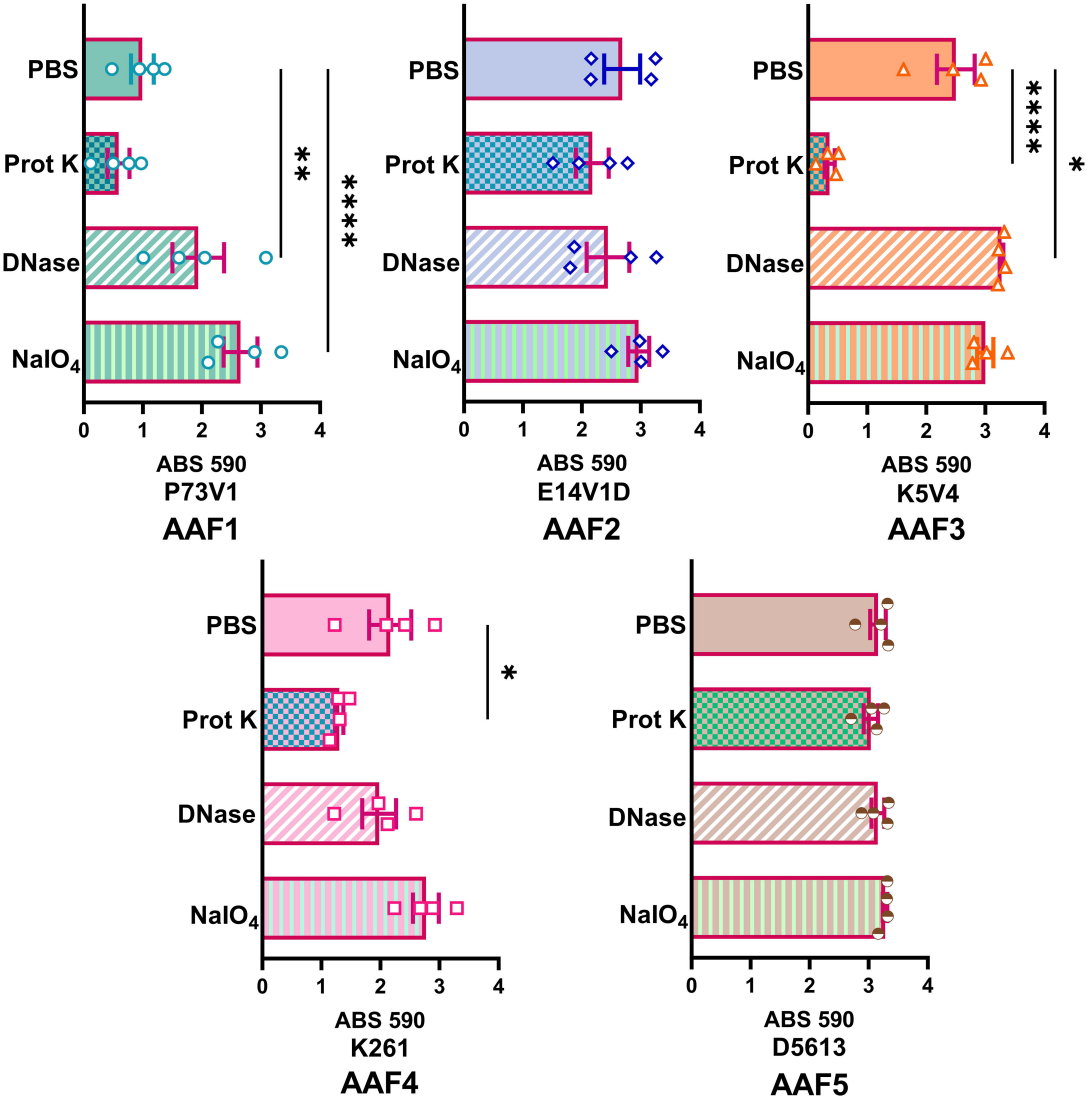
Quantitative staining of mature biofilms treated with DNase, proteinase K or sodium metaperiodate. After 18 hours of biofilm growth, the media was replaced with 1 mg/ml proteinase K (prot K) 1 mg/ml DNase I (DNase), or 7.5mM sodium metaperiodate (NaIO_4_) or a vehicle control (PBS) for 1 hour. Each dot (biological replicate) represents the mean of four technical replicates. Error bars indicate SEM. Significance tested by 1-way ANOVA with Šídák correction. *P ≤ 0.05; **P ≤ 0.01; ****P ≤ 0.0001.

**Figure 7:**
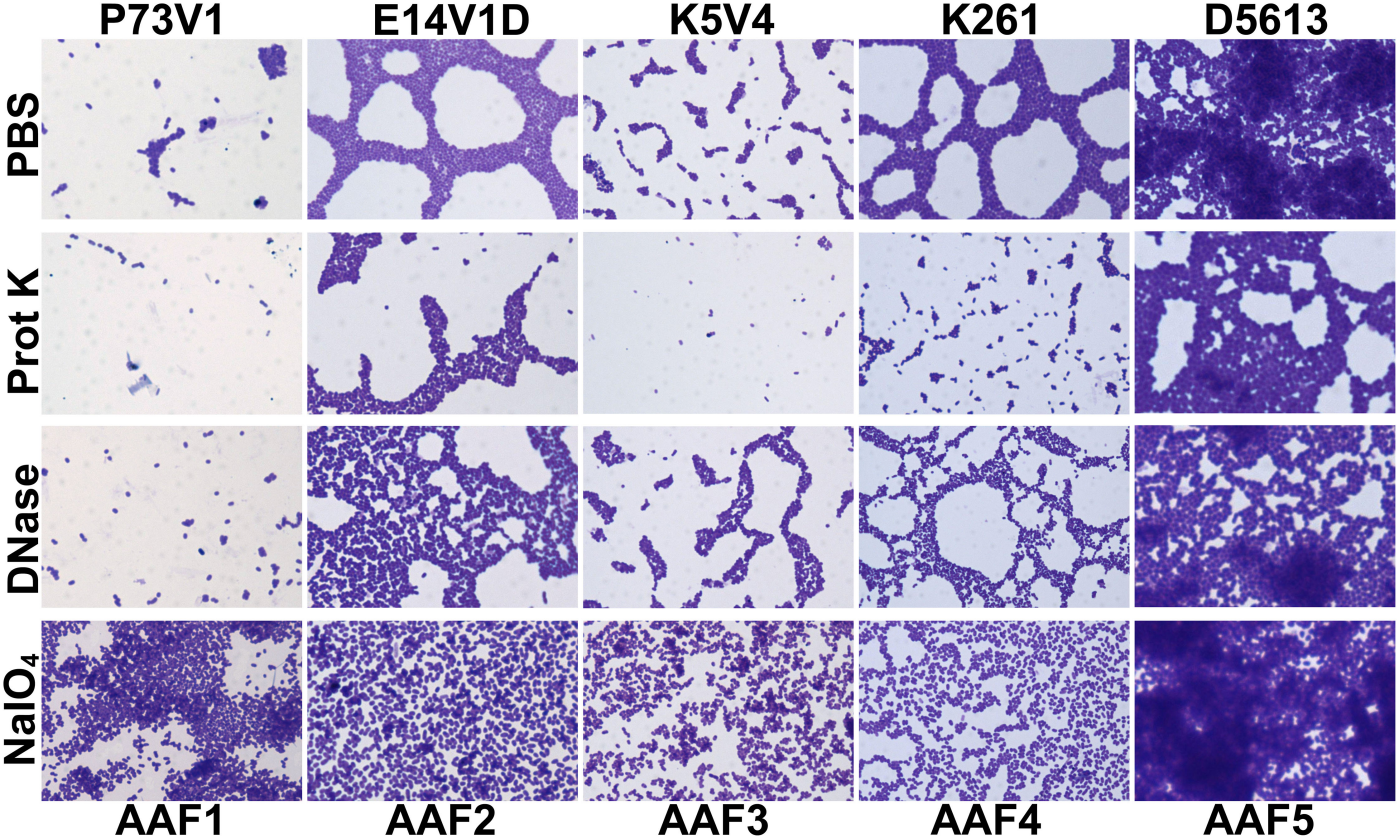
Images of mature biofilms treated with DNase, proteinase K or sodium metaperiodate. Biofilms were grown for 18 hours then media removed and replaced with 1 mg/ml proteinase K (prot K) 1 mg/ml DNase I (DNase), or 7.5mM sodium metaperiodate (NaIO_4_) or a vehicle control (PBS). After treatment for 1 hour at 37°C the biofilm disks were stained with crystal violet and imaged at 100x. Representative images were selected from two independent experiments.

To further elucidate differences in the extracellular components of biofilms from different EAEC strains, we next tested treating a mature biofilm with proteinase K, DNase, and sodium metaperiodate. For these studies we grew the biofilms for a shorter time, 18 hours, to determine if we could more easily detect changes to the biofilm structure. We also stained the biofilms with crystal violet, a nonspecific cationic stain, to better observe changes to the biofilm. We quantified biofilm staining after growth in 96-well plates (**Figure 6**) and took images of biofilms grown on disks (**Figure 7**). By comparing and contrasting the results of the qualitative and quantitative biofilm assay, we found a wide range of biofilm responses to the treatments as follows.

For P73V1 (AAF1), proteinase K exerted a minimal impact on the biofilm in both the quantitative staining and in the image. In contrast, DNase treatment led to a statistically significant increase in biofilm staining, but when the images were assessed, the result of the DNase treatment appears to be a breakup of the clumping of the bacteria (**Figures 6** and **7**). Sodium metaperiodate treatment also significantly increased the amount of biofilm stained and apparent in the image. The latter result was unexpected (**Figures 6** and **7**), because we thought cleavage of glycoproteins might lead to a reduction in biofilm formation.

For E14V1D (AAF2), we saw no significant change in the quantitative staining of the biofilm after the treatments (**Figure 6**). However, in the images we did note changes in the amount of clumping of the bacteria of the biofilm, with most of the treatments seeming to increase the distance among the bacteria (**Figure 7**). Yet, these apparent changes in biofilm images were not reflected by changes in quantitative biofilm staining (**Figure 6**).

The K5V4 (AAF3) proteinase K-treated biofilm had a strong reduction in quantitative staining and in the number of cells in the image (**Figures 6** and **7**). DNase treatment led to a slight increase in both biofilm quantitative staining and in the image. Sodium metaperiodate did not change quantitative biofilm staining but it did change the biofilm pattern, with cells appearing to be more spread out (**Figures 6** and **7**).

The strain K261 (AAF4) biofilm exhibited a statistically significant reduction in biofilm staining after proteinase K treatment, and the image of the proteinase K-treated biofilm reflected an apparent reduction in the number of bacteria (**Figures 6** and **7**). In contrast, although both DNase- and sodium metaperiodate-treated biofilms failed to show a significant change in biofilm quantitative staining, the images of the treated biofilms showed a breakup of the biofilm such that the bacteria appeared to be further apart (**Figures 6** and **7**).

Finally, the D5613 (AAF5) biofilm did not respond to any of the treatments, perhaps due to the large amount of biofilm staining and cells present (**Figures 6** and **7**).

**Figure 8:**
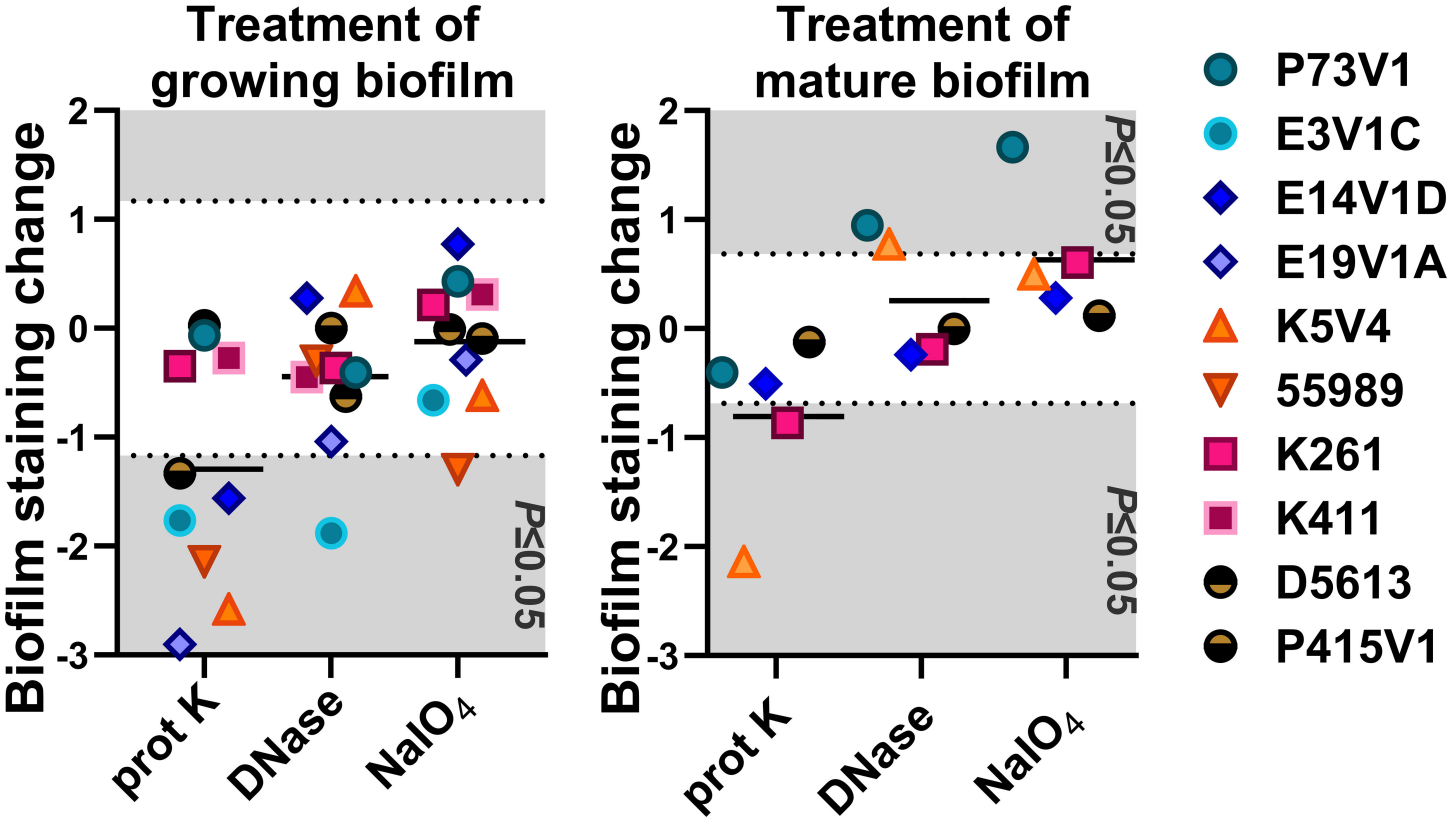
Normalized summary of all quantitative treatment results. In the left (treatment at the same time as biofilm formation) and right (treatment of mature biofilms) panels, we normalized the quantitative staining data by subtracting the after-treatment values from that of the sham-treated (PBS) control. The net change in staining is shown. Values from each bacterial strain are indicated by the color-coded symbols. Points that appear on the gray background are statistically significant changes (*P ≤* 0.05). The black line shows the average net change for all strains treated. Each point is the result of three or more independent experiments.

The most striking change we observed overall was that after sodium metaperiodate treatment there was an apparent increase in the number of cells in the images. A summary of all of the treatment results is shown in **Table 2**, and as normalized data in **Figure 8**.

**Table 2:** Summary of biofilm treatments^#^.

Fimbrial type/Strain name		During growth (Growing)							After growth (Mature)						
		Quantitative			Qualitative				Quantitative			Qualitative			
		prot K	DNase	NaIO_4_	PBS	prot K	DNase	NaIO_4_	prot K	DNase	NaIO_4_	PBS	prot K	DNase	NaIO_4_
AAF1	**P73V1**	**•**	**•**	**•**	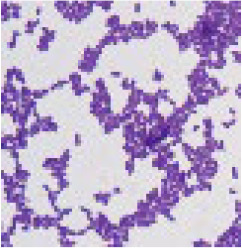	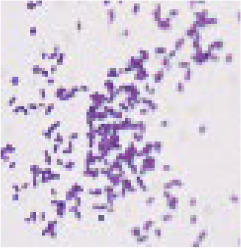	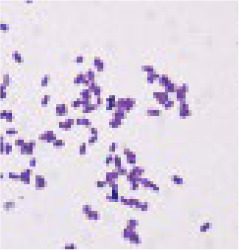	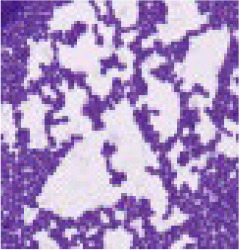	**•**	**↑↑**	**↑↑↑↑**	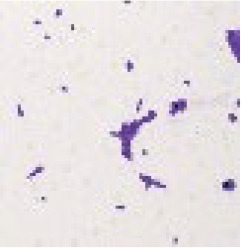	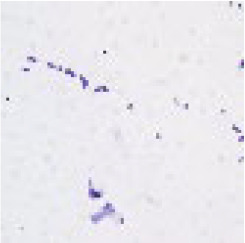	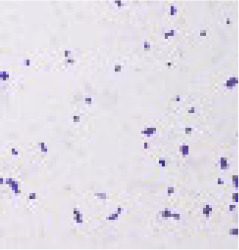	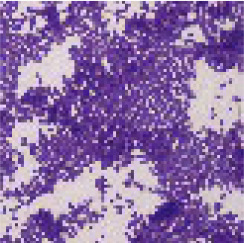
	**E3V1C**	**↓↓↓↓**	**↓↓↓↓**	**•**												
AAF2	**E14V1D**	**↓**	**•**	**•**	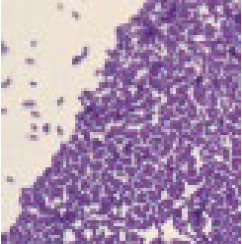	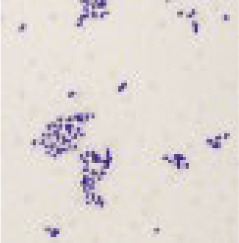	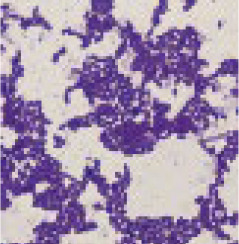	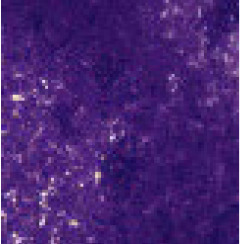	**•**	**•**	**•**	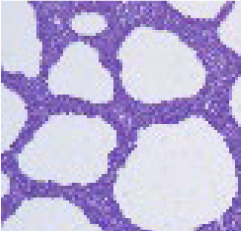	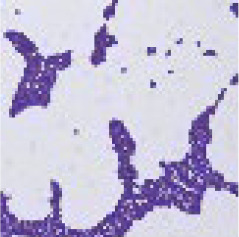	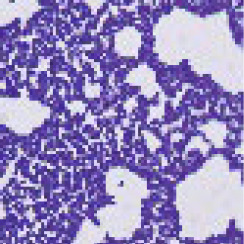	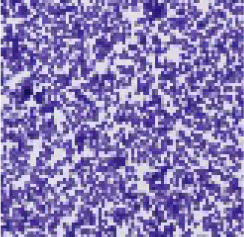
	**E19V1A**	**↓↓↓↓**	**•**	**•**											
AAF3	**K5V4**	**↓↓↓↓**	**•**	**•**	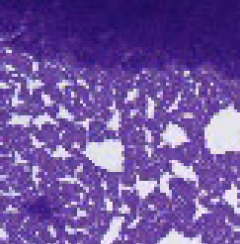	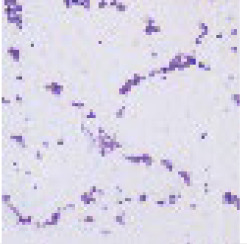	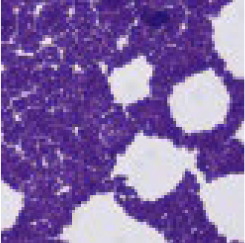	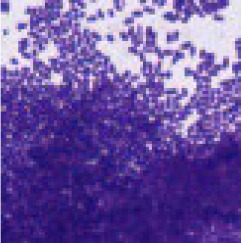	**↓↓↓↓**	**•**	**↑**	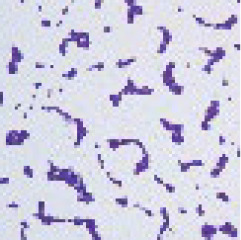	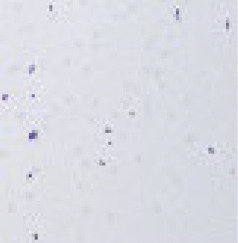	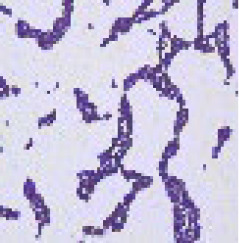	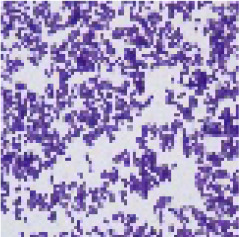
	**55989**	**↓↓↓↓**	**•**	**↓↓**											
AAF4	**K261**	**•**	**•**	**•**	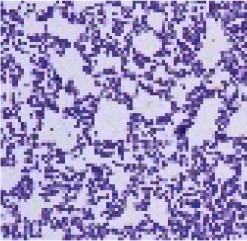	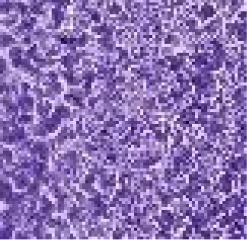	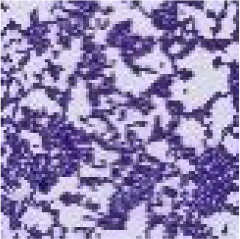	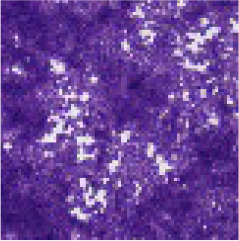	**↓**	**•**	**•**	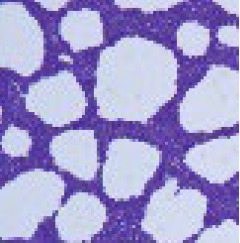	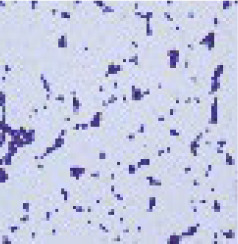	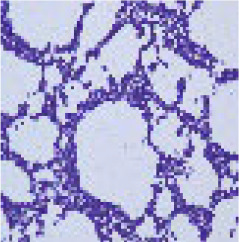	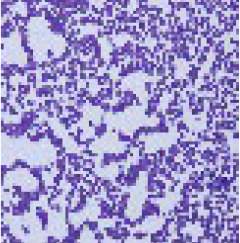
	**K411**	**•**	**•**	**•**											
AAF5	**D5613**	**↓**	**•**	**•**	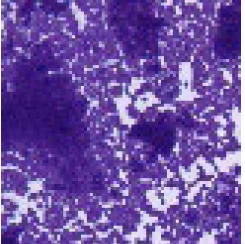	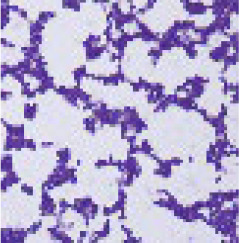	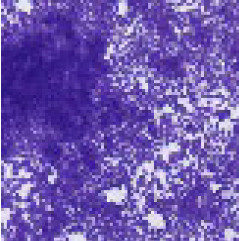	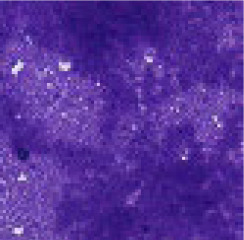	**•**	**•**	**•**	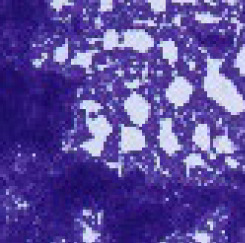	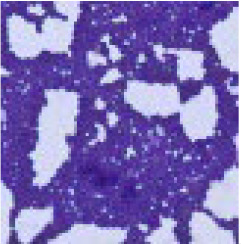	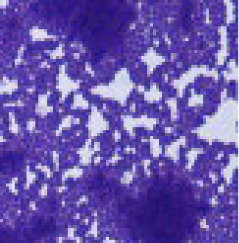	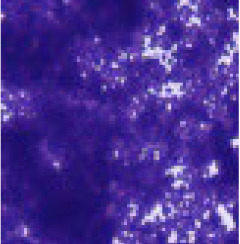
	**P415V1**	**•**	**•**	**•**											

^#^1 mg/ml proteinase K (prot K) 1 mg/ml DNase I (DNase), or 7.5 mM sodium metaperiodate (NaIO_4_). ↓*P* ≤ 0.05; ↓↓*P* ≤ 0.01; ↓↓↓*P* ≤ 0.001; ↓↓↓↓*P* ≤ 0.0001; •*P*>0.05; not significant.

## 3 Discussion

In this study, we found that quantitative biofilm staining did not correlate with AAF type, that overall, the EAEC biofilm appears similar in images, and that common treatments to target biofilms had limited effects. We explored the composition of the extracellular biofilm matrix of EAEC by several methods. Fluorescent stains were used to localize protein, eDNA, and glycoprotein within the biofilm. The results of those staining studies showed that EAEC strains have similar biofilm composition, and that all of the targets (protein, eDNA, glycoprotein) are present in the EAEC extracellular biofilm matrix. Overall, glycoprotein staining was less prevalent than eDNA and protein staining. The lower level of glycoprotein staining we observed might be due to the selective nature of the WGA conjugate we used. We did note that two strains, E3V1C (AAF1) and 55989 (AAF3), which had a high level of quantitative biofilm staining, showed more compact biofilm in the images. We do not know the reason for the distinction, but suspect that the nature of those two biofilms may allow for higher levels of crystal violet staining. Overall though, we were surprised that we did not observe more differences among the EAEC strains because of the differences in biofilm staining levels among the strains (**Figure 1**) and heterogeneity in the virulence factors such as adhesins (**Table 1**). We found the extracellular matrix of EAEC to be primarily associated with the individual cells, and that the matrix did not appear to extend out very far from the bacteria. We had hoped to observe a complex 3D structure of biofilm with confocal imaging. Instead, we found a mostly flat biofilm of one to two bacterial cell layers thick on the glass disks. This result could be due to the static growth conditions, which may allow weaker cell-to-cell attachment than a biofilm formed in the human gut which is subject to a strong flow.

We added treatments that target protein, eDNA, or carbohydrate into the medium with a growing biofilm to determine if such treatments would reduce biofilm staining. We expected to find that treatments that target protein, eDNA, or carbohydrate would reduce the staining of the extracellular component they targeted, i.e. that DNase would reduce the amount of TOTO-1 iodide staining, reflecting a reduction in eDNA. However, we observed no apparent reduction in matrix components in biofilm images when those treatments were used in the growth medium. In contrast, we did see changes in the arrangement of the EAEC cells in some images. For example, the E19V1A (AAF2) and 55989 (AAF3) biofilms treated with proteinase K had apparently fewer cells than the PBS control-treated biofilms in images (**Figure 3**). The reason for the apparent discrepancy may be due to the proteinase K detaching cells from the biofilm off of the glass disks rather than altering the biofilm matrix. For the growing biofilm, it may also be that if a treatment targets one component of the extracellular matrix, the bacteria are able to compensate by overproducing other matrix components. For the quantification of biofilms grown with the treatments, we found that 6/10 strains showed a reduction in biofilm formation with proteinase K treatment. This result demonstrates that protein does play a role for the developing biofilm for most, though not all, strains. The effect of proteinase K treatment was also reflected in the crystal violet-stained images of the strains (**Figure 5**). However, the crystal violet-stained images revealed that even though sodium metaperiodate treatment did not alter quantitative biofilm staining for most strains, that treatment with that carbohydrate-cleaving agent caused rearrangement of the cells on the glass disks. Although some of the differences between the images and the quantitative data may be due to the different methodology used in the studies, taken together, the results indicate that to assess the EAEC biofilm, both quantitative and microscopic analyses are necessary.

We also asked if treatment with proteinase K, DNase, and sodium metaperiodate could affect a fully-formed EAEC biofilm. However, with those single treatments, we did not generally measure a reduction in quantitative staining, despite the abundant presence of those components as detected by fluorescent staining (**Figure 6**). These results strongly suggest that the protein, eDNA, and carbohydrates are protected within the biofilm. The only exceptions were that treatment with proteinase K reduced quantitative biofilm staining for two of EAEC strains tested (K5V4 AAF3 and K261 AAF4), while DNase treatment increased biofilm staining for two strains (P73V1 and K5V4). For P73V1 (AAF1), sodium metaperiodate treatment also increased quantitative biofilm staining (**Figure 6**).

We expected to find that mature, fully-formed biofilms would be more resistant than a developing biofilm to the treatments tested. However, we found that some biofilms showed greater resistance while growing (P73V1 AAF1, K261 AAF4), and others were more resistant as a mature biofilm (E14V1D AAF2, D5613 AAF5). These findings indicate that the treatments have different effects depending on the state of the biofilm. It is possible that the permeability of the biofilm matrix may differ for a growing biofilm than for a mature biofilm. For example, D5613 (AAF5), which has high biofilm staining, was not impacted by any post-treatment but with during-growth treatment, proteinase K reduced biofilm staining (**Figure 4**). Finally, we found that although DNase treatment increased biofilm staining for two of five strains (P73V1 AAF1 and K5V4 AAF3) with fully mature biofilms (**Figure 6**), the biofilm from those same strains did not show a change when DNase was added to the developing biofilm (**Figure 4**). In the image of the DNase-treated mature biofilms from P73V1 (AAF1) and K5V4 (AAF3), it is possible that the change in the biofilm structure after DNase treatment led to increased retention of crystal violet stain, since the quantitative assays measure total bound crystal violet (**Figure 7**).

Assessing the treatments as a whole (summarized in **Figure 8** and **Table 2**), we found that proteinase K was the most likely treatment to cause a statistically significant change in biofilm staining. In contrast, DNase added during biofilm growth and on a mature biofilm on average did not alter staining in a statistically significant way.

Sodium metaperiodate treatment caused mostly non-significant effects as demonstrated by quantitative data, although the images clearly demonstrated a rearrangement of the EAEC within the biofilm. We initially hypothesized that the differences observed after sodium metaperiodate treatment might be due to an increase of simple sugars freed from cleaved polysaccharides, providing more energy for biofilm growth. However, the arguments against that hypothesis are that 1) Dulbecco’s Modified Eagle Medium (DMEM) media is already a high glucose medium with 4.5 g/L of d-glucose, 2) the post-growth treatment was only for 1 hour, a time period that would only allow for a few doublings, and, 3) sodium metaperiodate treatment did not alter the CFU/mL for planktonic cells, as tested under identical conditions (**Supplementary Figure 2**). Instead, we posit that the sodium metaperiodate catalyzes changes to the polysaccharides which leads to increased spacing around each cell without reducing the total number of cells, and allowing for increased uptake of crystal violet. These observations support the hypothesis that the sodium metaperiodate interacts with the biofilm by breaking down polysaccharide.

Overall, we found that crystal violet was a better stain than the fluorescent dyes for capturing visible differences in structure among the different biofilms, perhaps due to the relatively two-dimensional nature of the biofilms in these *in vitro* assays. The crystal violet-stained biofilms exhibited a notable lacy architecture. We are not sure whether the lacy pattern reflects the natural growth pattern of the biofilm or was partly influenced by the drying process that occurs prior to staining.

Our biofilm treatment results are similar to those published by Lim et al. for an O157:H7 enterohemorrhagic *E. coli* strain, for which DNase had minimal impact and proteinase K caused a significant reduction of the biofilm (Lim et al., 2019). In contrast, other non-EAEC *E. coli* biofilms are impacted by DNase and proteinase K, including visual changes to the biofilm (Tetz et al., 2009; Tetz and Tetz, 2010). DNase is also a strong inhibitor of biofilm formation for the unrelated bacteria *Pseudomonas aeruginosa*, *Streptococcus pneumoniae*, and *Staphylococcus aureus* (Whitchurch et al., 2002; Hall-Stoodley et al., 2008; Deng et al., 2022). Proteinase K causes a significant reduction for *Staphylococcus aureus* and *Helicobacter pylori* biofilms, but unlike for EAEC, sodium metaperiodate caused no visual alteration to the biofilm (Beltrame et al., 2015; Shukla and Rao, 2017; Windham et al., 2018).

Our findings indicate that the EAEC biofilm is complex and contains protein, DNA, and carbohydrate, and further, suggest that a single treatment is unlikely to eliminate an EAEC biofilm. In addition, our results indicate that different EAEC may require different treatments for successful biofilm elimination, though we acknowledge that these findings will need to be assessed under *in vivo* conditions. Finally, in this study we demonstrate that AAF type does not dictate biofilm staining level, and that the finding of low biofilm staining does not predict higher susceptibility to biofilm treatments.

## 4 Materials and methods

### 4.1 Strains

Most EAEC isolates came from the Trial Evaluating Ambulatory Therapy of Travelers’ Diarrhea (TrEAT-TD) study (Riddle et al., 2017; Petro et al., 2020). For an additional AAF5 strain, Nadia Boisen kindly provided D5613, (C267-15) from a recent Mozambique pediatric case (Boisen et al., 2020). 55989 is a prototypic EAEC with AAF3 (Mossoro et al., 2002).

### 4.2 Biofilms grown in 96-well plates

EAEC strains were grown in a shaking incubator at 37°C, normalized by optical density (OD), and diluted in Dulbecco’s Modified Eagle Medium (DMEM) high glucose with L-glutamine (Genesee Scientific 25-501) to 10^7^ CFU/mL and added to a 96-well flat-bottom untreated plate (VWR 82050-760). DMEM media was used as the control for each plate to subtract out background absorbance. After covering the plate with a lid, the plate was incubated at 37°C without shaking.

After incubation, the biofilm was washed once with phosphate-buffered saline (PBS) (Fisher Scientific 70-011-044) before fixing with ethanol for 10 minutes. Fixed biofilms were stained with a mixture of 3 mM crystal violet (Sigma Aldrich C0775) and 5% ethanol, and then rinsed with water and dried. The bound crystal violet was eluted with ethanol, and the absorbance was read at 590 nm.

### 4.3 Biofilm grown on disks

EAEC biofilms were inoculated on glass cover slips (Fisher 12-545-81P) in a 24-well plate in DMEM following the same conditions as for the 96-well plates. After-growth biofilms were treated as described above. Five fields of view were taken with an Olympus BX60F-3 under 100x oil immersion.

### 4.4 Treatment with DNase, proteinase K or sodium metaperiodate

#### 4.4.1 After/mature biofilm treatment

Biofilms were grown for 18 hours then the media removed and replaced with 1 mg/ml DNase (Sigma Aldrich DN-25), 1 mg/ml proteinase K (Fisher Scientific BP1700) or 7.5 mM sodium metaperiodate (Fluka Analytical 71859) or a vehicle control, PBS. After incubating for 1 hour at 37°C, the biofilm treatment was removed, and the biofilms fixed and stained with crystal violet or fluorescent stains.

#### 4.4.2 During growth biofilm treatment

Biofilms were grown for 18 hours with DMEM media supplemented to 1 mg/ml DNase, 1 mg/ml proteinase K or 7.5 mM sodium metaperiodate or equivalent volume of vehicle control (PBS).

### 4.5 Fluorescent staining for confocal microscopy

We adapted the staining method from Schaffer et al. (Schaffer et al., 2023). Filmtracer Sypro Ruby Biofilm Matrix (Fisher F10318) was used for proteins with 450/610 nm (excitation/admission) and red channel in images. For eDNA, we used cell-impermeable TOTO-1 iodide (Fisher T3600) 514/533 nm and the green channel. Because there is no universal stain for polysaccharides, we selected wheat germ agglutinin (WGA), a lectin that mainly targets *N*-acetylglucosamine and sialic acid residues. WGA has been used to stain other *E. coli* (Vogeleer et al., 2016). WGA conjugate CF640R (Biotium 29026-1) 642/662 nm and colored in images as violet. As a counterstain we used Hoechst 33342 (Sigma Aldrich B2883) which stains all DNA 350/461 nm, blue in images.

Fixed glass disks were stained in the 24-well plate, with Sypro Ruby for 20 minutes, 1:100,000 TOTO-1 iodide for 3 minutes, 2μg/mL WGA conjugate for 15 minutes, and 1 μg/mL Hoechst 33342 for 5 minutes, washing once with water after each stain. The disk was fixed overnight in 2% paraformaldehyde. Each disk was removed and glued to a glass slide with Cytoseal XYL (Thermo Scientific 8312-4) and imaged at 64x oil immersion on a Zeiss LSM 980. Five areas were randomly selected for each of the stained disks for imaging. Representative single layer images and composite 3D images were exported using ZEN lite 107.8 software (RRID: SCR_023747). The fluorescent intensity cannot be quantified for comparison due to differences in settings used to capture the images.

### 4.6 Statistical analysis

We used GraphPad Prism 10.0.3 (RRID: SCR_002798) to test significance of three or more biological replicates with the test and multiple comparison correction listed in each figure legend *P ≤ 0.05; **P ≤ 0.01; ***P ≤ 0.001; ****P ≤ 0.0001.

## Data availability statement

The original contributions presented in the study are included in the article/**Supplementary Material**. Further inquiries can be directed to the corresponding author.

## Author contributions

VV: Conceptualization, Data curation, Formal analysis, Investigation, Methodology, Visualization, Writing – original draft. AM: Conceptualization, Data curation, Funding acquisition, Project administration, Supervision, Writing – review & editing.

## Author disclaimer

The opinions and assertions expressed herein are those of the authors and do not reflect the official policy or position of the Uniformed Services University of the Health Sciences or the Department of Defense. References to non-Federal entities or products do not constitute or imply a Department of Defense or Uniformed Services University of the Health Sciences endorsement. The opinions and assertions expressed herein are those of the authors and do not reflect the official policy or position of the Henry M. Jackson Foundation for the Advancement of Military Medicine, Inc. Neither the author nor any family members have a financial interest in any commercial product, service, or organization providing financial support for this research.
