# Supplementary material for: Extracellular components in enteroaggregative Escherichia coli biofilm and impact of treatment with proteinase K, DNase or sodium metaperiodate
Source: Front Cell Infect Microbiol. 2024 May 29;14:1379206. doi: 10.3389/fcimb.2024.1379206 (PMC11209426; doi:10.3389/fcimb.2024.1379206)
Supplement: Supplementary file 1 [file DataSheet_1.docx]

***Supplementary Material***

# Supplementary Figures and Tables

## Supplementary Figures

**Supplementary Figure 1. 3D-composite image of biofilms visualized with fluorescent stains**. Biofilms grown on glass disks for 25 hours were fixed and stained with Filmtracer Sypro Ruby Biofilm Matrix (red) for most classes of proteins; TOTO-1 iodide (green), a cell-impermeant DNA stain; WGA conjugate (violet) for glycoproteins; and Hoechst (blue) for total DNA as a bacterial counter stain. Z-stacks were rendered in 3D using ZEN lite 107.8 software. Representative fields of view were selected from two independent experiments.

**Supplementary Figure 2. Incubation with enzymes and sodium metaperiodate did not alter bacterial viability**. Planktonic cells of the indicated strains were incubated for one hour with the indicated treatments at the same concentration as the biofilm assays. Each dot represents a biological replicate. Significance was tested by one-way ANOVA with Šídák correction. ns – not significant, *P* > 0.05.
