# Supplementary figures and images for: Extracellular components in enteroaggregative Escherichia coli biofilm and impact of treatment with proteinase K, DNase or sodium metaperiodate
Source: Front Cell Infect Microbiol. 2024 May 29;14:1379206. doi: 10.3389/fcimb.2024.1379206 (PMC11209426; doi:10.3389/fcimb.2024.1379206)

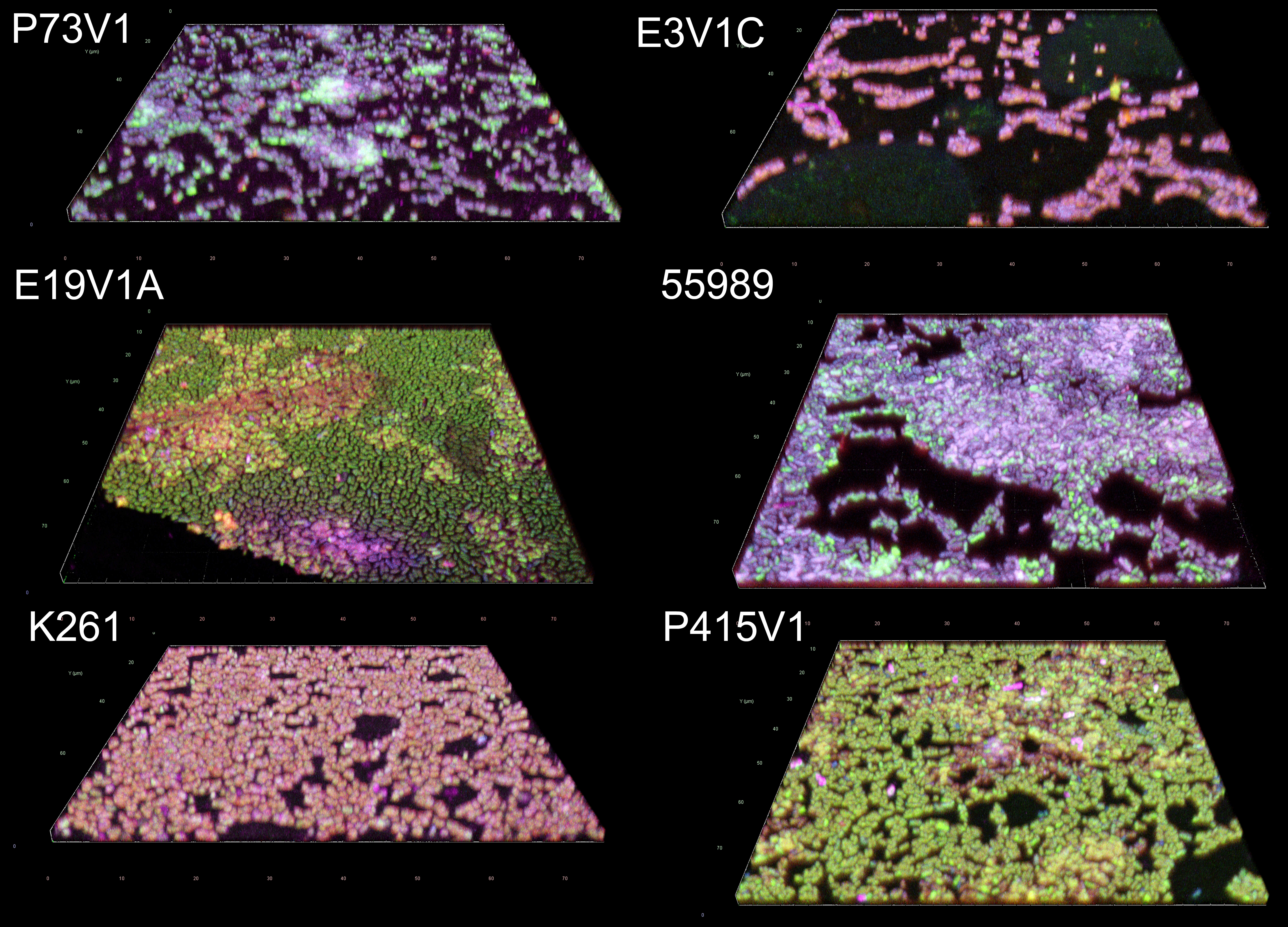

Supplement: Supplementary file 2 [file Image_1.jpeg]

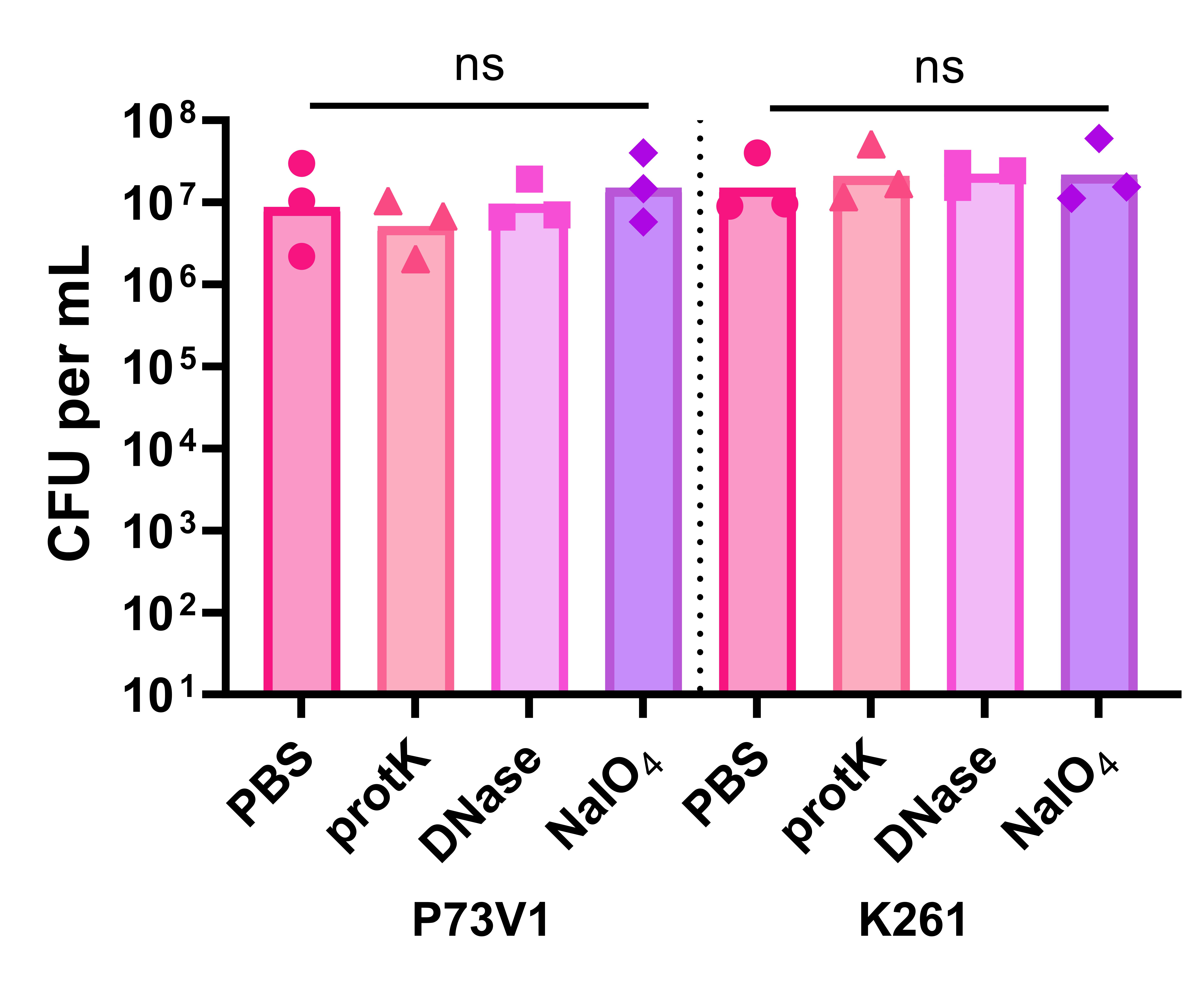

Supplement: Supplementary file 3 [file Image_2.jpg]
